# Dehydroandrographolide inhibits mastitis by activating autophagy without affecting intestinal flora

**DOI:** 10.18632/aging.103312

**Published:** 2020-07-23

**Authors:** Wenjin Guo, Juxiong Liu, Yufei Zhang, He Ma, Yuhang Li, Qian Gong, Yu Cao, Guiqiu Hu, Shengnan Xie, Shoupeng Fu

**Affiliations:** 1 College of Veterinary Medicine, Jilin University, Changchun 130062, China

**Keywords:** dehydroandrographolide, mastitis, autophagy, AMPK, intestinal flora

## Abstract

Mastitis can seriously damage the physical and mental health of lactating women. The use of antibiotics and anti-inflammatory drugs may damage the flora balance in lactating women. To alleviate mastitis in lactating women and reduce drug-induced damage to the flora, we found that dehydroandrographolide (Deh) has good anti-inflammatory and bacterial balance functions. *In vivo*, we found that Deh significantly inhibited the expression of MPO, IL6, IL-1β, TNF-α, COX2 and iNOS and reduced pathological damage to the mammary gland. The feces in the control and Deh groups were collected and sequenced for 16S flora. The results showed that Deh did not change the primary intestinal microflora composition of the two groups. *In vitro*, our study showed that Deh significantly inhibited the expression of *IL6, IL-1β* and *TNF-α* in the EpH4-Ev cell line. When an AMPK inhibitor was added, the anti-inflammatory effect of Deh was blocked. To further study the anti-inflammatory mechanism of Deh, we found that Deh significantly promoted autophagy through the phosphorylation of AMPK, Beclin and ULK1. In conclusion, our study found that Deh promoted autophagy and played an anti-inflammatory role by activating the AMPK/Beclin/ULK1 signaling pathway and did not affect intestinal flora.

## INTRODUCTION

Mastitis is a female disease that easily develops during lactation [1]. Breast milk contains a variety of nutrients for the growth and development of the newborn, and it is very beneficial for the digestion and absorption of the baby [2]. Mastitis seriously damages the physical and mental health of women and affects breastfeeding [3]. If treatment is not timely, the development of mastitis is even life-threatening [4]. Mastitis is usually caused by the interaction of pathogenic microorganisms, the environment, nutrition and organism functional states [5–7]. Among them, pathogenic microorganisms are the main factor that causes mastitis [8]. Most pathogens, especially *Escherichia coli*, cause acute clinical mastitis with obvious clinical symptoms [9]. Lipopolysaccharide (LPS) is the main component of the cell wall of gram-negative bacteria and activates many types of cells (macrophages and epithelial cells) to produce proinflammatory mediators [10–12]. LPS has been widely used to establish inflammatory models *in vivo* and *in vitro* [13]. In addition, the mastitis model of mice established by injecting LPS into the mammary duct has similar symptoms to those caused by *E. coli* infection [14, 15]. Moreover, the LPS-induced mastitis model has been widely used to screen anti-mastitis drugs [16].

Dehydroandrographolide (Deh) is an extract of andrographolide [17] that has antibacterial, anti-inflammatory and antiviral effects [18–21]. However, the effect of Deh on intestinal flora has not been reported. The intestinal flora is closely related to human health [22]. There are a large number of probiotics in the intestinal flora to maintain health [23]. Therefore, it is important to maintain the normal intestinal microbial balance in the treatment of mastitis [24]. This study focused on Deh as the research object and used LPS to establish a mouse mastitis model. We observed and verified the effect of Deh on mastitis and intestinal flora in mice and further explored the mechanism of Deh on EpH4-Ev cells (a normal mouse mammary epithelial cell line) through *in vitro* experiments. These findings support the screening of leading compounds with anti-inflammatory effects in the future.

## RESULTS

### Dehydroandrographolide (Deh) alleviates mammary injury in LPS-induced mastitis mice

**Figure 1:**
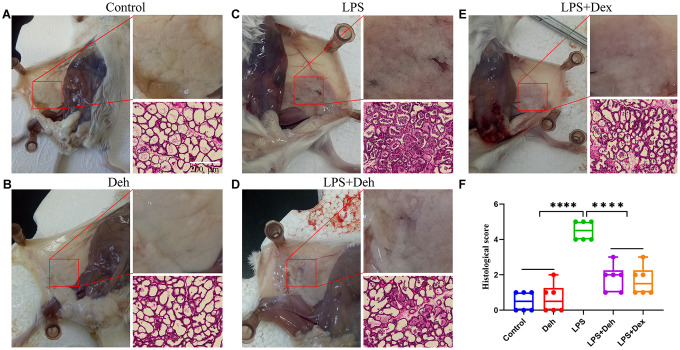
**Dehydroandrographolide (Deh) alleviates LPS-induced mammary injury in mice.** (**A**–**E**) The histological effect of Deh on LPS-induced mastitis in mice and the effect of Deh on neutrophil infiltration in the mouse mammary gland. (**F**) Histological score of the mouse mammary gland. The values are presented as the mean ± SD (*p<0.05, **p<0.001, ***p<0.001 and ****p<0.0001).

First, we took pictures of the mammary glands and observed damage to the mammary glands in mice. Then, we collected the mammary glands and stained them with H&E. Dexamethasone (Dex) was used as a positive control to effectively alleviate mastitis [11]. The results showed that the mammary glands of the control and Deh groups had no pathological changes (Figure 1A, 1B), while those of the LPS group had obvious edema and hyperemia (Figure 1C). The pathological symptoms of the LPS + Deh group and LPS + Dex group were significantly improved (Figure 1D–1E). The H&E results were also consistent with the previous results. There was no neutrophil infiltration in the Control or Deh groups (Figure 1A, 1B). In the LPS group, there was substantial neutrophil infiltration in the acini (Figure 1C). In the LPS + Deh and LPS + Dex groups (Figure 1D, 1E), neutrophil infiltration in the acini was significantly decreased. The histological scores showed that Deh significantly alleviated LPS-induced mouse mastitis, which was consistent with the results of the positive control group (Figure 1F).

### Deh reduces the inflammatory response in the mammary glands of mastitis mice

**Figure 2:**
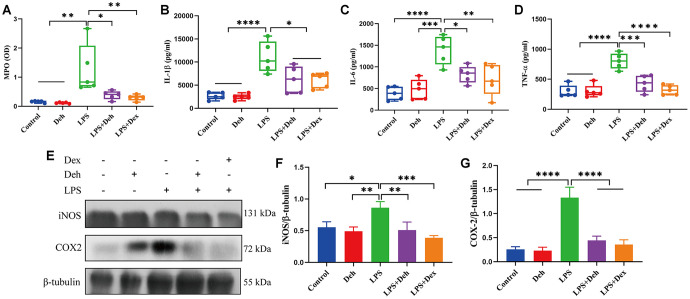
**Deh inhibits the expression of COX2, iNOS, IL-6, IL-1β and TNF-α in mastitis mice.** (**A**) MPO activity in the mammary gland. (**B**–**D**) The protein expression of IL-6, IL-1β and TNF-α. (**E**, **G**) Protein levels of COX2 and iNOS. The values are presented as the mean ± SD (*p<0.05, **p<0.001, ***p<0.001 and ****p<0.0001).

MPO, COX2, iNOS, IL-6, IL-1β and TNF-α are important indicators of inflammation. Our results showed that the MPO activity and the protein levels of COX2, iNOS, IL-6, IL-1β and TNF-α in the mammary glands of the LPS group were significantly higher than those in the LPS + Deh and LPS + Dex groups (Figure 2A–2G). This result indicates that Deh significantly reduces the inflammatory response of the mammary gland.

### Sequencing quality, microbial abundance and microbial differences

The number of effective tags in the control and Deh groups was in accordance with the following experimental standards (Supplementary Figure 1A, 1B). The Shannon curve results showed that the curve of each sample was flat, and the amount of sequencing data was sufficient (Supplementary Figure 1D). The results of the rank abundance curve showed that the species composition between each group of samples was rich and uniform (Supplementary Figure 1C). The PCA and PCoA results showed that the microbial composition of each mouse was similar (Supplementary Figure 1E, 1F).

### Deh does not disrupt the intestinal microbiological balance

**Figure 3:**
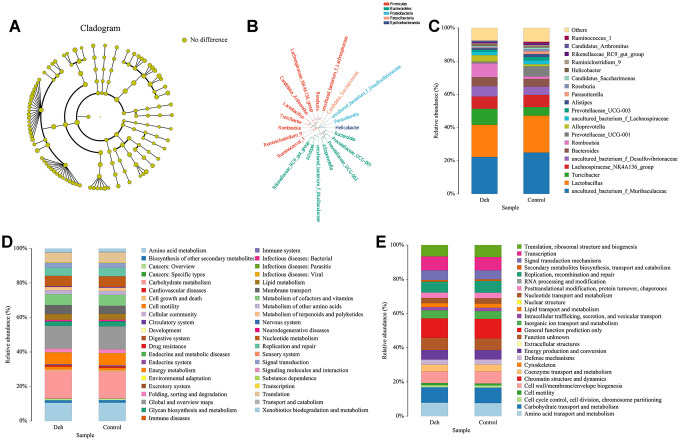
**Effect of Deh on the composition of intestinal flora.** (**A**) Line discriminant analysis between the control and Deh groups. (**B**) Phylogenetic tree of the species. (**C**) The composition of intestinal flora in the control and Deh groups. (**D**, **E**) Functional genes and metabolic pathways in the control and Deh groups.

**Figure 4:**
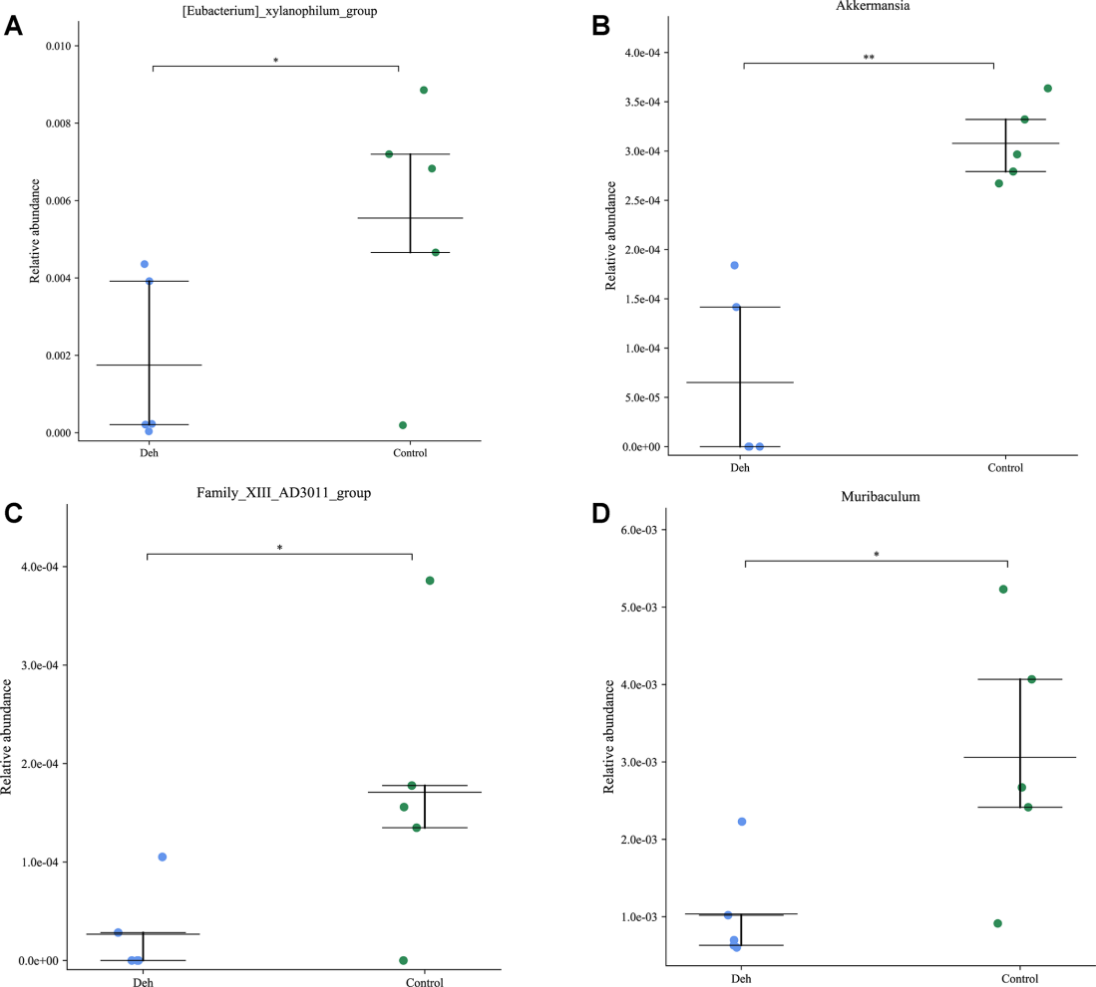
**Effect of dehydroandrographolide on low abundance flora.** (**A**–**D**) The levels of Family XIII AD3011 group, [Eubacterium] xylanophilum group, Akkermansia and Muribaculum in the control and Deh croups. The values are presented as the mean ± SD (*p<0.05, **p<0.001, ***p<0.001 and ****p<0.0001).

The line discriminant analysis (LDA) effect size results showed that there were no significantly different microbial groups among the different groups (Figure 3A). Figure 3B is the phylogenetic tree of the species. The species distribution histogram showed that the relative species content between the two groups did not change much, and Deh did not change the relative species content (Figure 3C). The results of KEGG and GO analysis showed that there was no significant difference in functional genes and metabolic pathways between the two groups (Figure 3D, 3E). Although Deh did not change the main structure of the intestinal flora, it did affect the levels of some flora. At the genus level, the abundances of the family XIII AD3011 group, [Eubacterium] xylanophilum group, Akkermansia and Muribaculum in the Deh group were lower than those in the control group (Figure 4A–4D). At the species level, Parabacteroides sp. YL27, [Eubacterium] xylanophilum group, Family XIII AD3011 group and Akkermansia in the Deh group were lower than those in the Control (Supplementary Figure 2A–2D).

### Effect of Deh on cell survival

**Figure 5:**
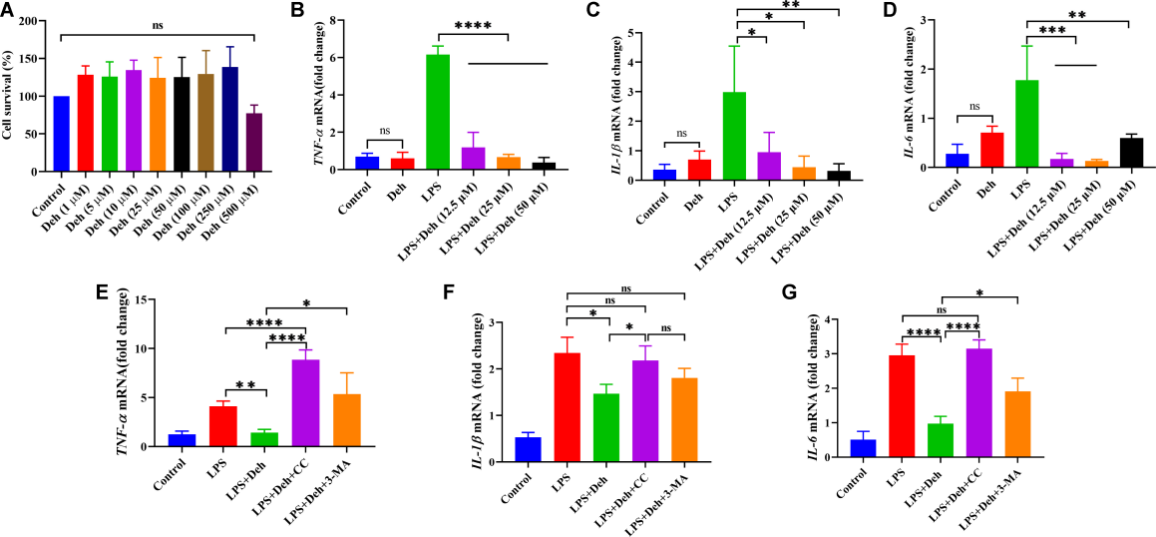
**Deh inhibits the gene expression of *IL-6, IL-1β* and *TNF-α* in EpH4-Ev cells.** (**A**) Effect of Deh on the survival of EpH4-Ev cells. (**B**–**G**) Gene expression of *IL-6, IL-1β* and *TNF-α.* The values are presented as the mean ± SD (*p<0.05, **p<0.001, ***p<0.001 and ****p<0.0001).

CCK8 assay results showed that Deh concentration did not affect cell activity when it was less than 250 μM. At 500 μM, Deh decreased cell activity, but there was no significant difference (Figure 5A).

### The effect of Deh on IL-6, IL-1β and TNF-α in LPS-induced EpH4-Ev cells

The qRT-PCR results showed that Deh did not cause inflammation in EpH4-Ev cells. Moreover, the gene levels of *IL-6, IL-1β* and *TNF-α* in the Deh + LPS group were significantly lower than those in the LPS group (Figure 5B–5D). The Deh-mediated inhibition of *IL-6, IL-1β* and *TNF-α* was concentration dependent. After adding an AMPK inhibitor, we found that Deh did not inhibit *IL-6, IL-1β* and *TNF-α* (Figure 5E–5G). Compared with the levels in the LPS + Deh group, *IL-1β* and *TNF-α* in the LPS + Deh + 3-MA group increased significantly, and *IL-6* also increased (Figure 5E–5G). These results indicate that Deh inhibits the LPS-induced inflammatory response in EpH4-Ev cells through the AMPK/autophagy signaling pathway.

### Deh promotes autophagy through the AMPK/Beclin/ULK1 pathway in EpH4-Ev cells

**Figure 6:**
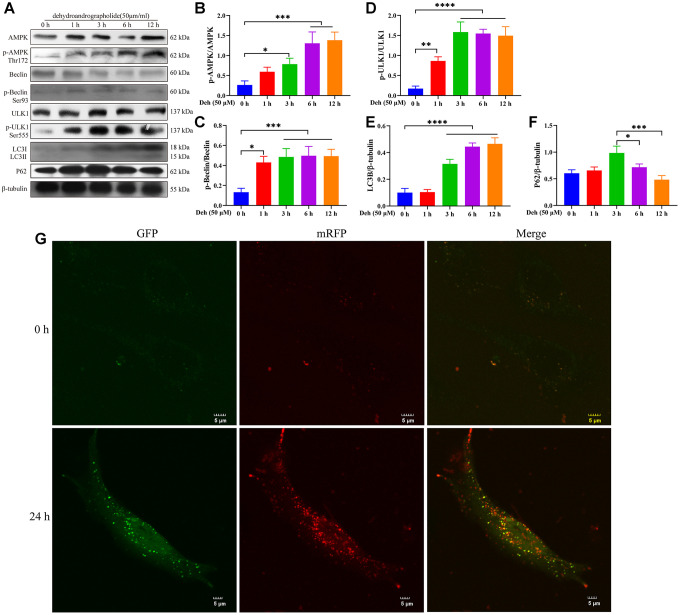
**Deh promotes autophagy by phosphorylating AMPK, Beclin and ULK1.** (**A**–**F**) Protein levels of p-AMPK, AMPK, p-Beclin, Beclin, p-ULK1, ULK1, LC3B and P62. (**G**) Effects of Deh on autophagic flux at 0 and 24 h. The values are presented as the mean ± SD (*p<0.05, **p<0.001, ***p<0.001 and ****p<0.0001).

**Figure 7:**
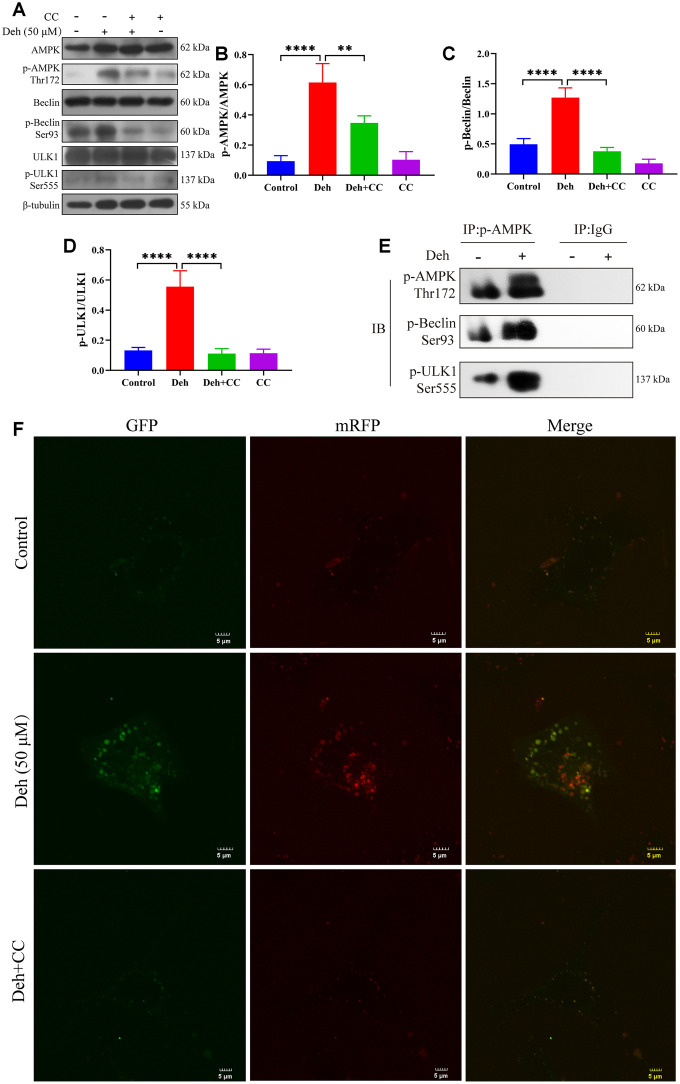
**Deh activates autophagy through AMPK.** (**A**–**D**) Protein levels of p-AMPK, AMPK, p-Beclin, Beclin, p-ULK1 and ULK1. (**E**) The interaction of p-AMPK with p-ULK1 and p-Beclin. (**F**) Deh affects autophagic flux through AMPK. The values are presented as the mean ± SD (*p<0.05, **p<0.001, ***p<0.001 and ****p<0.0001).

Previous studies have shown that p-AMPK promotes autophagy through phosphorylation of AMPK, Beclin and ULK1. Autophagy plays an important role in inhibiting the inflammatory response. To further study the anti-inflammatory mechanism of Deh, we first detected whether Deh activated autophagy through AMPK signaling in EpH4-Ev cells. Our results showed that Deh significantly promoted the phosphorylation of AMPK, Beclin and ULK1 at 3 h, 6 h and 12 h (Figure 6A–6D). Moreover, Deh significantly promoted the degradation of p62 and increased LC3B (Figure 6E, 6F). The immunofluorescence results showed that autophagy was highest in the Deh group at 24 h (Figure 6G). We further detected the protein levels of p-AMPK, p-Beclin and p-ULK1 after adding the AMPK inhibitor CC. The results showed that the protein levels of p-AMPK, p-Beclin and p-ULK1 in the Deh + CC group were significantly lower than those in the Deh group (Figure 7A–7D); Deh did not enhance the autophagic flux of EpH4-Ev cells (Figure 7F). In addition, the Co-IP results suggested that Deh significantly promoted the phosphorylation of AMPK, while the phosphorylation of AMPK significantly enhanced the phosphorylation of Beclin and ULK1 (Figure 7E). These results showed that Deh activates autophagy by AMPK.

### The effect of Deh on autophagy in the EpH4-Ev cell inflammatory response model

**Figure 8:**
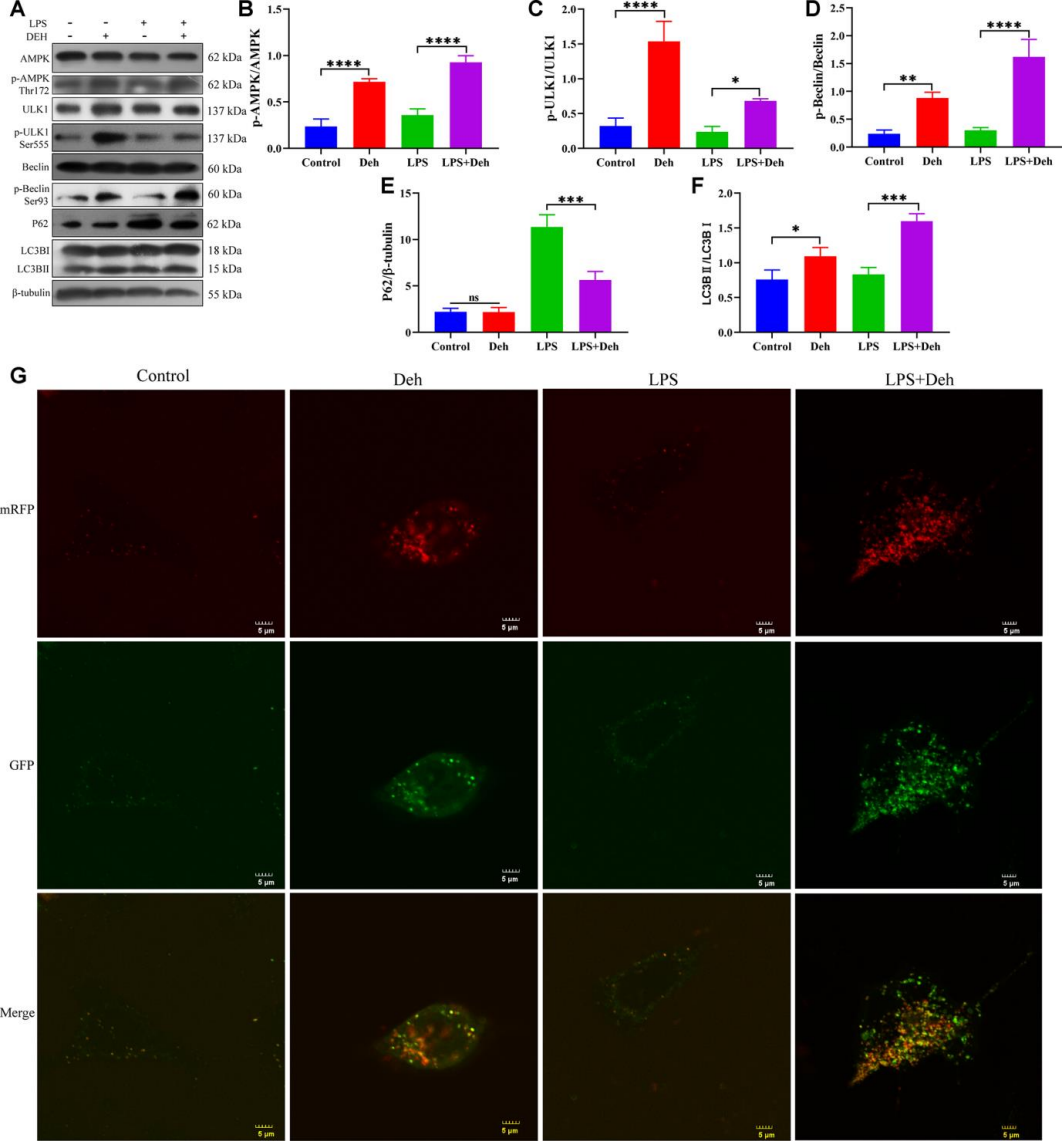
**Deh activates autophagy in the EpH4-Ev cell inflammatory response model.** (**A**–**F**) Protein levels of p-AMPK, AMPK, p-Beclin, Beclin, p-ULK1, ULK1, LC3B and P62. (**G**) Effect of Deh on autophagic flux in the EpH4-Ev cell inflammatory response model. The values are presented as the mean ± SD (*p<0.05, **p<0.001, ***p<0.001 and ****p<0.0001).

Previous results showed that Deh activates autophagy through the AMPK signaling pathway [21]. To verify that autophagy plays an important anti-inflammatory role in Deh, we next tested whether Deh promoted autophagy in an inflammatory response model of EpH4-Ev cells. We detected the protein levels of p-AMPK, p-Beclin, p-ULK1, P62, LC3B and autophagic flux in the EpH4-Ev cell inflammatory response model. The results showed that the protein levels of p-AMPK, p-Beclin, p-ULK1 and LC3B in the Deh and Deh + LPS groups were significantly upregulated, and p62 was significantly downregulated (Figure 8A–8F). The autophagic flux in the Deh and Deh + LPS groups was also significantly enhanced (Figure 8G).

## DISCUSSION

Our study showed that Deh significantly alleviates mastitis, improves LPS-induced mammary damage, and does not damage the intestinal flora balance. *In vitro*, we found that Deh plays an anti-inflammatory role by activating autophagy through AMPK phosphorylation.

Natural products are mainly natural animal and plant extracts [25]. Several studies have shown that many natural products have anti-inflammatory and antibacterial functions [26, 27]. Li et al. found that farrerol improves mastitis through the ERK1/2, P38 and AKT signaling pathways [28]. Kan et al. also found that myricetin alleviates mastitis and enhances the blood-milk barrier [14]. In the present study, H&E staining and observation of mammary tissue showed that LPS seriously damaged the mammary gland. Deh significantly improved the congestion, edema and neutrophil infiltration of the mammary gland. Some studies have shown that MPO is an important marker of the inflammatory response that directly reflects the severity of the inflammatory response. Moreover, LPS increased COX2, iNOS, IL-6, IL-1β and TNF-α in the mammary gland. The increase in proinflammatory mediators in the mammary gland exacerbates mastitis and causes secondary damage to the mammary gland. Our results showed that Deh significantly inhibited the expression of COX 2, iNOS, IL-6, IL-1β and TNF-α. These results suggest that Deh alleviated LPS-induced mastitis by reducing the release of proinflammatory mediators.

Some studies have shown that anti-inflammatory drugs or antibacterial drugs may change the intestinal flora, destroy the balance in the flora and cause other serious consequences [29, 30]. Becattini et al. showed that antibiotics cause intestinal flora damage and disease [31]. Silverman et al. found that antibiotic treatment changed the composition and metabolic function of intestinal microflora, which may be related to necrotizing enterocolitis and antibiotic-associated diarrhea [32]. Antibiotics cause the destruction of intestinal flora, and steroidal or non-steroidal anti-inflammatory drugs destroy the balance of intestinal flora. Steroids cause anxiety or depression by changing the structure of the flora [33, 34]. Otani et al. found that non-steroidal anti-inflammatory drugs lead to changes in the microbial population and cause intestinal damage [35]. These studies showed that although the use of antibiotics and anti-inflammatory drugs alleviate bacterial infection and inflammation, the destruction of intestinal flora by these drugs also leads to other serious consequences. In particular, taking antibiotics or anti-inflammatory drugs for a long time will seriously damage the balance of intestinal flora. In our study, we found that Deh did not cause changes in the abundance or composition of the primary flora, but there were significant changes in the species and genera of four bacteria. Among them, xylanophilum is positively related to the occurrence of small cell lung cancer. It has also been reported that *Muribaculum* has been detected more in colorectal cancer, gastric cancer and Crohn's disease [36]. Our sequencing results showed that Deh significantly reduced the levels of *xylophilum* and *Muribaculum*. This suggests that Deh does not disrupt the flora balance and may reduce the risk of colitis.

Although we have preliminarily described the anti-inflammatory effect of Deh and its effect on intestinal flora *in vivo*, the mechanism of its anti-inflammatory effect *in vivo* is still unclear. Wenbi Xiong et al. found that Deh upregulates hBD-2 to enhance the innate immunity of the intestine [20]. Our study found that Deh significantly activated the AMPK signaling pathway and promoted the phosphorylation of AMPK. Previous studies have shown that the activation of AMPK promotes autophagy by phosphorylating Beclin and ULK1 [37, 38]. In complex multicellular organisms, autophagy proteins, which are the core molecular mechanism of autophagy, coordinate the different responses of cells and tissues to other dangerous stimuli, such as infection [39, 40]. Recent developments have revealed the important role of autophagy pathways and proteins in immunity and inflammation [41, 42]. Autophagy balances the beneficial and harmful effects of immunity and inflammation and prevents infectious, autoimmunity and inflammatory diseases [42]. However, autophagy can be divided into classical and nonclassical pathways [43]. Our study showed that Deh activated the nonclassical pathway of autophagy. Interestingly, the results showed that Deh phosphorylated AMPK, Beclin and ULK1 to enhance autophagy, which depends on the activation of AMPK. We found that Deh promotes the formation of autophagic flux over time.

In conclusion, our study showed that Deh activates autophagy through AMPK to play an anti-inflammatory role and that Deh does not affect the balance of major intestinal flora.

## MATERIALS AND METHODS

TRIzol, LPS and phenylmethanesulfonyl fluoride (PMSF) were purchased from Sigma (Saint Louis, MO, USA). Deh was purchased from Shanghai Yuanye Bio-Technology Co. CCK8 was purchased from Saint-Bio Co. (Shanghai, China). Compound C (CC) and 3-methyladenine (3-MA) were purchased from Selleckchem (Shanghai, China). p-Beclin, Beclin, p-ULK, ULK, AMPK, p-AMPK and LC3B were purchased from Cell Signaling Technology (Boston, MA, USA). P62 was purchased from Proteintech (Rosemont, IL, USA). β-Tubulin was purchased from Bosterbio in Dallas (Pleasanton, USA). IgG was purchased from Beyotime (Shanghai, China). HRP-conjugated anti-mouse and anti-rabbit secondary antibodies were purchased from Bosterbio.

### Animal experiments

**Figure 9:**
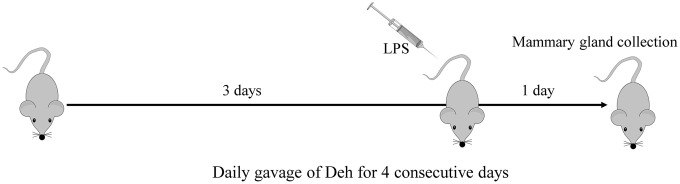
**Construction of the mouse mastitis model.**

ICR mice (8~10 weeks old, 25~30 g weight) were purchased from the Center of Experimental Animals of Baiqiuen Medical College of Jilin University (Jilin, China). The animals were housed in certified, standard laboratory cages and administered food and water *ad libitum* before experimental use. All animal care and experimental procedures were conducted in accordance with the guidelines established by the Jilin University Institutional Animal Care and Use Committee (approved on 27 February 2015; Protocol No. 2015047) designated ‘Guide for the Care and Use of Laboratory Animals’ and approved by the Institutional Animal Care and Use Committee of Jilin University. Animal studies were performed in compliance with ARRIVE guidelines. Lactating mice (5~7 days after birth of offspring) were randomly divided into five groups: control (n=6), Deh (100 mg/kg), LPS (0.2 mg/mL, 50 μL) treatment (n=6), LPS + Deh (100 mg/kg) (n=6), and LPS+Dex (5 mg/kg) (n=6). On day four of lactation, the control group was fed normal saline, and the two treatment groups were fed Deh (100 mg/kg) for 4 days. On day 3, LPS was injected into the fourth inguinal mammary gland of the mice. At 24 h after LPS injection, the mice were anesthetized with sodium pentobarbital (45 mg/kg), and the mammary glands were collected (Figure 9).

### Cell culture

EpH4-Ev cells were purchased from American Type Culture Collection (ATCC^®^ CRL-3063™) and were cultured in DMEM containing 10% FBS at 37°C in a humidified incubator with 5% CO_2_.

### Cell viability

The effect of Deh on cell viability was determined using a CCK8 assay. EpH4-Ev cells were treated with Deh (1 μM, 5 μM, 10 μM, 25 μM, 50 μM, 100 μM, 250 μM, and 500 μM) for 4 h. Subsequently, 10 μL CCK8 was added to each well. After 1 h, the absorbance (OD) was measured at 450 nm on a microplate reader (Bio-Rad, CA, USA).

### Plasmids and fluorescence microscopy

Cultured cells were seeded in 24-well plates with microscope cover slips (Thermo Fisher Scientific) and were transfected with mRFP-GFP-LC3 (a gift from the Pathology Laboratory, College of Veterinary Medicine, Jilin University) using LipoFiter (HANBIO) for 24 h. After the designated treatments, the cells were fixed with 4% paraformaldehyde (PFA) in PBS. All cellular images were obtained using an inverted confocal microscope (dissecting the dynamic turnover of GFP-LC3 in the autolysosome).

### Histological evaluation of the mammary gland

The mammary glands of mice were photographed and evaluated histologically. Then, for histological analysis of the mammary gland, the mice were euthanized, and the four pairs of mammary glands were fixed in 4% paraformaldehyde, followed by dehydration with ethanol. After paraffin embedding, 6 μm sections were cut and stained with hematoxylin and eosin (H&E) according to a previously described protocol [44]. Direct visual observation of the mammary gland and H&E-stained sections were examined under a light microscope to evaluate pathological changes. Simultaneously, a standard assessment method was conducted to determine mammary gland injury. Overall mammary gland injury was scored based on edema, neutrophil infiltration and hemorrhage, and three visual fields were observed for each slice. Studies were performed in a blinded manner. Injury scores were representative of severity (0, no damage; 1, mild damage; 2, moderate damage; 3, severe damage; and 4, very severe damage) [45].

### Protein levels of TNF-α, IL-6 and IL-1β

The protein levels of TNF-α, IL-6 and IL-1β in mammary glands were determined by ELISA kits according to the manufacturer’s instructions.

### Myeloperoxidase (MPO) activity assay

Mammary glands were collected and weighed, and MPO was analyzed according to a previously described protocol [45]. Samples were measured for MPO activity with a microplate reader at OD_450_.

### qRT-PCR analysis

Total RNA was isolated from cultured mMECs with TRIzol reagent (Invitrogen, Carlsbad, CA, USA), and amplification reactions were performed to detect the gene levels of *TNF-α, IL-6* and *IL-1β* [46]. The primer sequences are shown in Table 1.

**Table 1:** The primer sequences of *TNF-α, IL-1β*, *IL-6* and *β-actin.*

**Gene**	**Primer**	**Length (bp)**
*TNF-α* (sense)	5’-ACGGCATGGATCTCAAAGAC-3’	116
*TNF-α* (antisense)	5’-GTGGGTGAGGAGCACGTAGT-3’	
*IL-1β* (sense)	5’-GCTGCTTCCAAACCTTTGAC-3’	121
*IL-1β* (antisense)	5’-AGCTTCTCCACAGCCACAAT-3’	
*IL-6* (sense)	5’-CCGGAGAGGAGACTTCACAG-3’	134
*IL-6* antisense)	5’-CAGAATTGCCATTGCACAAC-3’	
*β-actin* (sense)	5’-GTCAGGTCATCACTATCGGCAAT-3’	147
*β-actin* (antisense)	5’-AGAGGTCTTTACGGATGTCAACGT-3’	

### Western blotting

Total proteins were isolated from EpH4-Ev cells and mouse mammary glands with RIPA lysis buffer (Beyotime, Shanghai, China) (50 mM Tris, pH 7.4; 150 mM NaCl; 1% Triton X-100; 1% sodium deoxycholate; 0.1% SDS; sodium orthovanadate; sodium fluoride; EDTA, leupeptin; and 1 mM PMSF). Tissue lysates were centrifuged at 12000 g for 5 min at 4 °C, and protein concentrations were determined with a Pierce^TM^ BCA protein assay kit (Thermo Scientific, China). Equal amounts of cell (30 μg)/mammary gland (50 μg) extracts were subjected to 12% sodium dodecyl sulfate-polyacrylamide gel electrophoresis (SDS-PAGE) and subsequently transferred to PVDF membranes (Millipore, Darmstadt, Germany) for antibody blotting. The membranes were incubated with primary antibodies (1:2000 dilution) at 4°C overnight, followed by HRP-conjugated goat anti-mouse (1:10000) or goat anti-rabbit secondary antibodies (1:10000) at room temperature for 1 h. Protein bands were visualized using a Beyo Enhanced Chemiluminescence Reagent Kit (Beyotime, Shanghai, China) according to the manufacturer’s instructions.

### Coimmunoprecipitation (Co-IP)

We scraped the cells off the dish with a cell scraper and then washed them once with PBS. Then, ice-cold IP lysis/wash buffer (250 μL) was added to the cells. The suggested amount of total protein per IP reaction was 500–1000 μg, as determined by a Pierce BCA protein assay (Thermo-Fisher, Rockford, USA). The Co-IP assay was conducted using a Pierce^TM^ Classic Magnetic IP/Co-IP Kit [46].

### Fecal sample collection and 16S sequencing

Fecal samples were collected from the mouse colons and stored at -80 °C. Fecal DNA and the V4-V5 region of the bacterial 16S ribosomal RNA gene were extracted and detected by BioMaKer (Beijing, China). The data analysis of sequencing results was also completed by BioMaKer.

### Statistical analysis

Images were generated using GraphPad Prism software (La Jolla, CA, USA). Animals were randomly assigned to groups. In mouse studies, histological analysis was conducted in a blind manner. In cases where overall F-tests were significant (*P* < 0.05), post hoc comparisons using Tukey’s method of adjustment were conducted to determine the location of significant pairwise differences. Analyses were performed using GraphPad Prism 8.02 software.

## Supplementary Material

Supplementary Figures
