# Supplementary material for: Dehydroandrographolide inhibits mastitis by activating autophagy without affecting intestinal flora
Source: Aging (Albany NY). 2020 Jul 23;12(14):14050–65. doi: 10.18632/aging.103312 (PMC7425474; doi:10.18632/aging.103312)
Supplement: Supplementary Figures [file aging-12-103312-s001..pdf]

SUPPLEMENTARY FIGURES

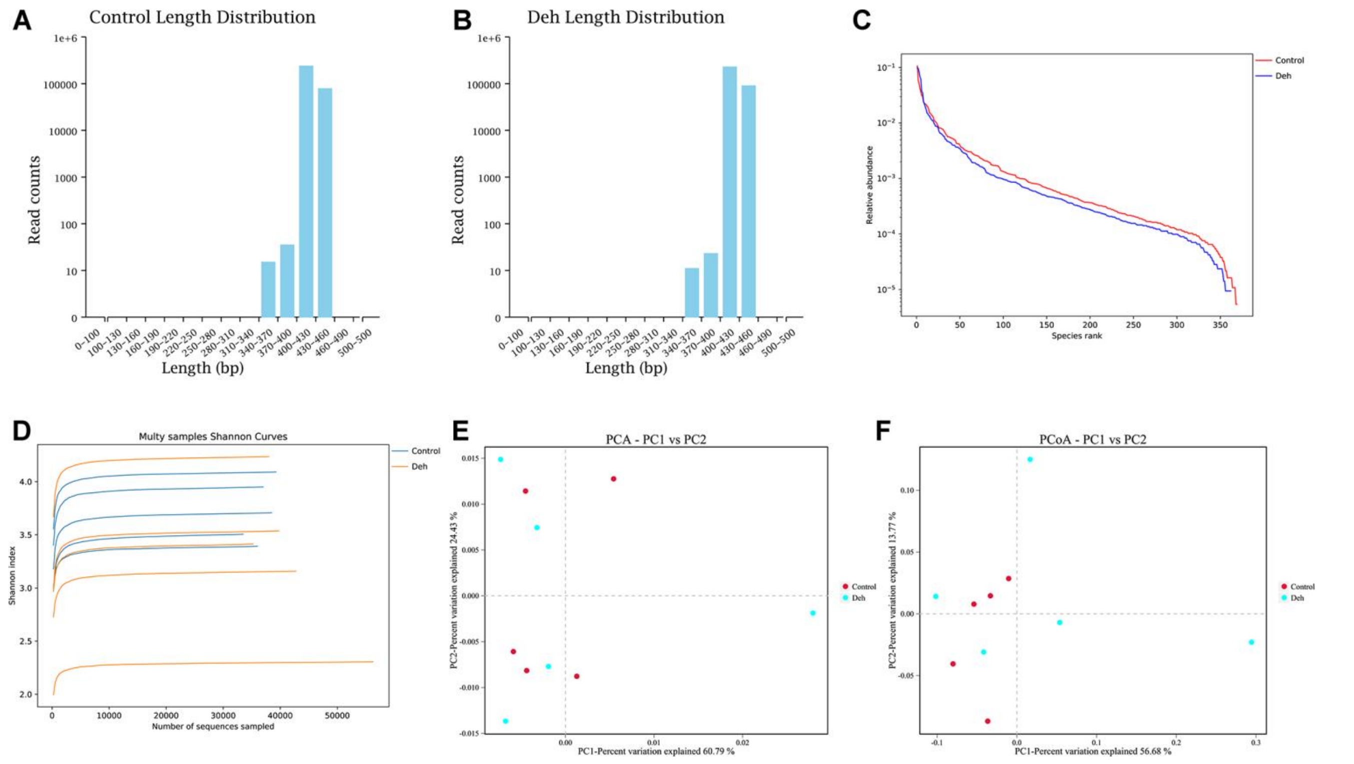

Supplementary Figure 1. The quality of microbiome sequencing in mice feces. (**A**, **B**) Length distribution in Control and Deh. (**C**) Relative abundance in Control and Deh. (**D**) Shannon index in Control and Den. (**E**, **F**) PCA and PCoA analysis between Control and Deh.

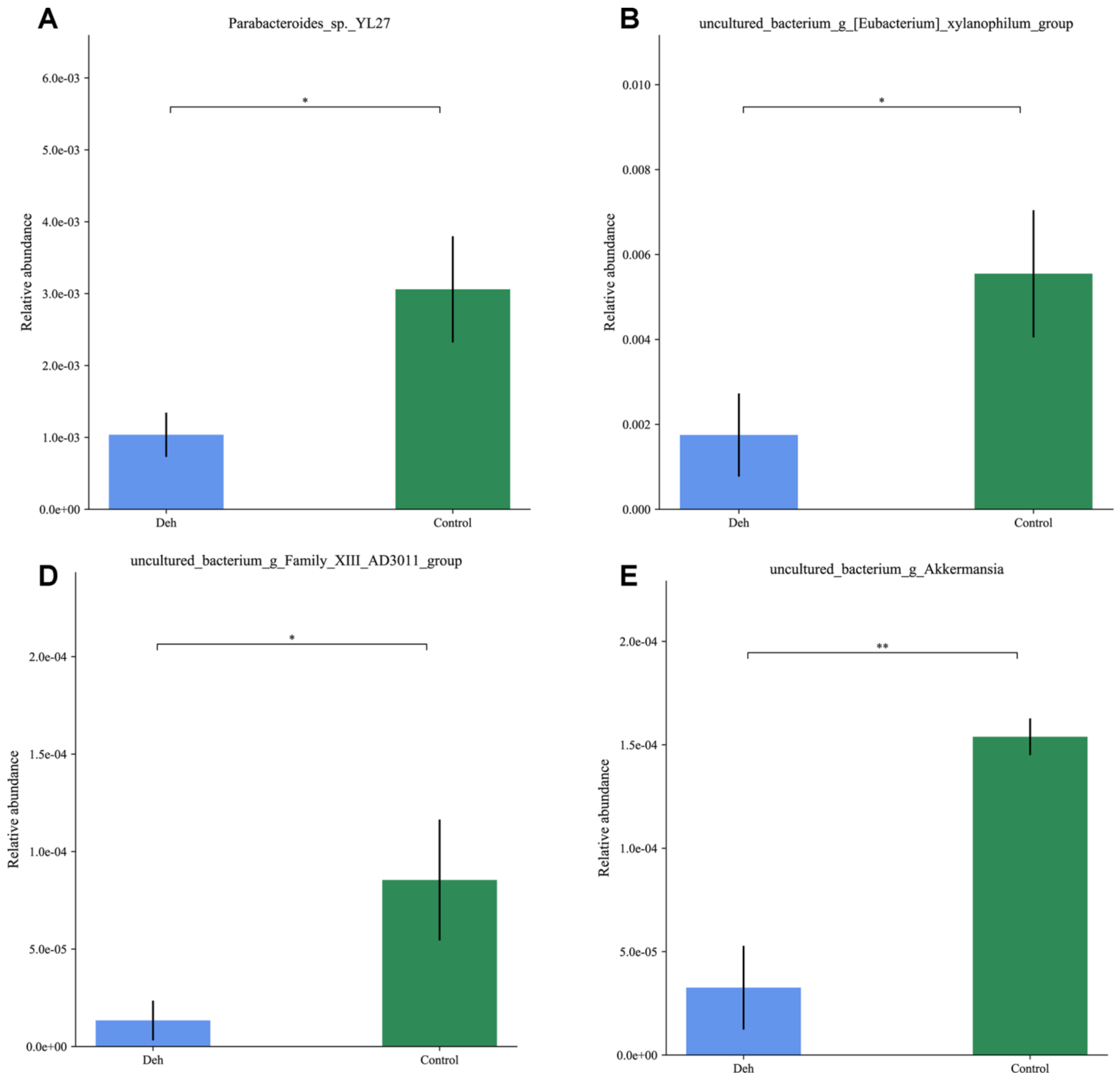

Supplementary Figure 2. Effect of dehydroandrographolide on low abundance flora. (**A**–**D**) At the species level, Parabacteroides sp. YL27, [Eubacterium] xylanophilum group, Family XIII AD3011 group and Akkermansia in Deh and Control group. Values are presented as the mean ± SD (**p*<0.05, ***p*<0.001, ****p*<0.001 and *****p*<0.0001)**.**
